# High Prevalence of Hepatitis C Virus Infection in Primitive Tribes of Eastern India and Associated Sociobehavioral Risks for Transmission: A Retrospective Analysis

**DOI:** 10.1089/heq.2019.0005

**Published:** 2019-11-04

**Authors:** Shantanu Kumar Kar, Jyotsnamayee Sabat, Lal M. Ho, Rasmi Arora, Bhagirathi Dwibedi

**Affiliations:** ^1^ Directorate of Medical Research, IMS and SUM Hospital, S “O” A University, BBSR, Bhubaneswar, India.; ^2^ Virus Research and Diagnostic Laboratory, Regional Medical Research Centre (ICMR), Bhubaneswar, India.; ^3^ Division of Enteric and Communicable Disease, Indian Council of Medical Research, New Delhi, India.; ^4^ Department of Pediatrics, AIIMS, Bhubaneswar, India.

**Keywords:** HCV, primitive tribe, risk factor

## Abstract

**Purpose:** The primitive tribal groups (PTGs) need special attention because of their low population growth: declining population size with high mortality rates. Scanty reports are available on the prevalence of hepatitis C virus (HCV) infection in primitive tribes of the country emphasizing their cultural and social practices associated with transmission of the disease.

**Methods:** The study was conducted on 1765 tribal individuals covering 5 PTGs, namely Lodha, Saora, Khadia, Juanga, and Mankidia, from 6 districts of Odisha. Serum samples were tested for the anti-HCV antibody using commercially available enzyme immunoassays. HCV RNA was detected based on the 5′ NCR region and genotyping was done by direct sequencing of the core region. Potential risk factors for HCV transmission were collected using a questionnaire and subjected to regression analysis through SPSS, version 17.0.

**Results:** Antibody to HCV was detected in 0%, 3.3%, 5.7%, 8.5%, and 13.4% in Saora, Lodha, Khadia, Juanga, and Mankidia tribes, respectively. HCV RNA was detected in 8.6% (11/127) of the samples tested. Genotyping of HCV isolates in all HCV RNA-positive samples revealed genotype 1b. Sharing of razors and shaving by the village barber were found to be significantly (*p*<0.05) associated with HCV transmission in males, whereas tattooing and multiple injections were found to be significant risk factors for females.

**Conclusion:** This study indicated a high prevalence of HCV infection in Mankidia and Juanga tribes in comparison with the national scenario, which needs public health attention.

## Introduction

With lack of prophylactic vaccination and cost-effective treatment, hepatitis C virus (HCV) has emerged as an important public health problem in India.^1–4^ Hence, identifying the population with high-risk behavior for HCV transmission and formulating strategies for prevention of transmission remain the mainstay of research.

The tribal communities in India are distinct in their sociocultural practices, socioeconomic developments that affect their morbidity pattern, and health-seeking behavior.

Limited studies are available on tribal communities in India reporting an antibody prevalence ranging between 1% and 13.7%,^5–10^ which includes primitive tribal groups (PTGs) from Andaman and Nicobar Islands and Madhya Pradesh. These are important information sources toward morbidity and mortality because of transmission risk related to community behavior and potential chronicity of liver disease. However, no report is available on this infection from the state of Odisha where a large proportion of the total population is represented by the tribal community (>22%), which includes 13 PTGs. PTGs are of greater concern because they are specially endangered groups with either declining or stagnant population growth and a very low level of literacy and social development indicated by the preagricultural level of technology.^11^

This study reports the first evidence on molecular epidemiology of HCV infection in five PTGs of Odisha and possible risk factors prevailing in these communities associated with HCV transmission.

## Methods

**FIG. 1.**
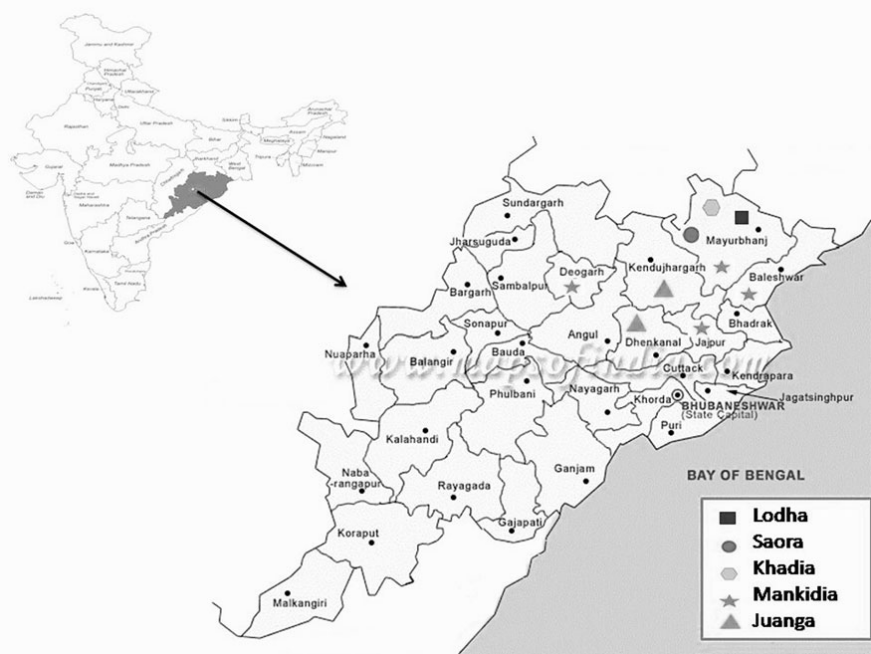
Distribution of PTGs in six districts of Odisha. PTGs, primitive tribal groups.

This community-based prevalence study from 2007 to 2009 included randomly selected 1765 subjects from 6 districts (Keonjhar, Mayurbhanj, Balasore, Bhadrak, Deogarh, and Jajpur) of Odisha covering 5 primitive tribes, Lodha (*n*=241), Saora (*n*=212), Khadia (*n*=450), Juanga (*n*=460), and Mankidia (*n*=401). The studied PTGs resided in hilly and subhilly locations of Similipal forest range and adjacent mountains in north and northeastern parts of the state (Fig. 1). The inhabitation areas were remote, inaccessible without round-the-year road communication, and the populations were living in isolation from the general community and health system. Tribal settlements or villages from each of the clustered tribal inhabitation areas were selected following the proportionate to size sampling procedure. Field survey was conducted by the team using door-to-door visits and staying in the area for a long period to establish community rapport for enrollment of subjects. Individuals of all ages and both sexes were included after obtaining informed written consent. Information on illness due to hepatitis infection, history of immunization, and risk factors for transmission was recorded.

Venous blood samples (4–5 mL) were collected aseptically from the individuals. Serum was separated, aliquoted in the field, and transported in liquid nitrogen to the laboratory. Because of remoteness of study areas, the samples could reach the center's laboratory in around 7 days.

Serological tests were undertaken to detect total antibodies to HCV using a third-generation ELISA (Ortho diagnostics, United Kingdom) kit. HCV RNA was extracted from patient's serum using a high-purity viral RNA extraction kit as per manufacturer's instructions (QIAGEN, Valencia, CA). Reverse Transcriptase Polymerase Chain Reaction (RTPCR) was carried out according to the procedure of Lole et al.^12^ for amplification of the 5′ NCR region of HCV.

For genotype identification, HCV RNA-positive samples were subjected to PCR amplification of the core region of the HCV genome.^12^ Sequencing was done by the Sanger dideoxy method,^13^ followed by amplicon sequencing using an automated DNA sequencer (Genetic Analyzer 3730; ABI). A phylogenetic tree was computed among genotypes of HCV using the Kimura 2-parameter matrix and neighbor-joining method in MEGA software, version 4.^14^

Quality control was assured by duplicate testing of all positive samples and 5% of negative samples at the National Institute of Virology, Pune, Maharashtra, India.

Information on potential risk factors and routes for transmission of HCV prevailing in the community was obtained through individual and group questionnaires.

The study was approved by the Institutional Review Board of the center and the Department of Tribal Welfare, Government of Odisha, before conducting the study.

Data were entered into Epi Info software and double-checked for entry errors. Statistical analysis was performed using 17.0 SPSS software. The prevalence of HCV infection was expressed as the percentage of seropositive samples against total subjects tested. Multiple logistic regression analysis was done to access the relative significance of possible risk factors. Odds ratio (OR) and confidence intervals of 95% (CI) were used to measure the strength of the association. A level of *p*<0.05 was used to indicate statistical significance.

## Result

Subjects who were enrolled (*n*=1765) in the study included all ages and both sexes. All subjects were screened for the presence of anti-HCV antibodies and 127 (7.1%) of them were positive. Tribe-wise prevalence was 0%, 3.3%, 5.7%, 8.47%, and 13.4% in Saora, Lodha, Khadia, Juanga, and Mankidia tribes, respectively (Table 1).

**Table 1.** Anti-Hepatitis C Virus Prevalence (%) in Different Age Groups

	Tribe wise prevalence/distribution				
Age group	Lodha (*n*=241), +ve (%)	Khadia (*n*=450), +ve (%)	Mankidia (*n*=401), +ve (%)	Juanga (*n*=460), +ve (%)	Total, +ve (%)
0–5	0 (0)	1 (3.8)	1 (1.9)	1 (2.6)	3 (11.11)
6–15	1 (12.5)	1 (3.8)	8 (14.8)	20 (51.3)	30 (23.6)
16–30	1 (12.5)	9 (34.6)^a^	16 (29.6)	6 (15.4)	32 (25.2)
31–45	3 (37.5)	9 (34.6)^a^	17 (31.5)^a^	6 (15.4)	35 (27.5)^a^
46–60	3 (37.5)	3 (11.5)	6 (11.1)	3 (7.7)	15 (11.8)
>60	0 (0)	3 (11.5)	6 (11.1)	3 (7.7)	12 (9.4)
Total	8 (3.3)	26 (5.7)	54 (13.4)	39 (8.4)	127 (7.1)

The Saora tribe has zero antibody prevalence, hence not included in the table.

^a^ *p*<0.05 (chi-square test comparing age-specific prevalence in the respective tribes); % prevalence was calculated, taking the No. of individuals tested in the respective age group as the denominator.

Antibody prevalence was increased with increasing age and observed to be high in the 31- to 45-year age group; however, in the Juanga tribe, higher prevalence was noted at 6 to 15 years of age (Table 1). Among 20 identified HCV positives in the 6–15-year age group from the Juanga tribe, 14 (5 females and 9 males) were <12 years old. Similarly, among the Mankidia tribe, 6 of 8 HCV positives were in the 6–15-year age group, 6 were <12 years old (3 females and 3 males).

An infection rate of 1.9% to 3.8% was seen in children <5 years of age in Khadia, Mankidia, and Juanga tribes. Overall prevalence of anti-HCV in males and females was 7.5% and 6.8%, respectively.

Among the samples seropositive for anti-HCV, viral RNA was detected in 8.6% of samples showing the anti-HCV antibody (*n*=11). Sequencing and phylogenetic analysis revealed circulation of genotype 1b.

Clinically overt hepatic disease was observed to be low (2.5%), and 140 (7.93%) individuals had past history of jaundice. Among HCV-positive individuals, 21.2% (27/127) had present and past history of liver disease.

The frequency distribution of risk factors for transmission of HCV in the respective tribal communities with respect to positive and negative anti-HCV status is given in Table 2. In the two tribes, that is, Mankidia and Juanga, having high prevalence of anti-HCV, the factors significantly associated with HCV infection were sharing of razors and shaving by the village barber. In the Mankidia tribe, among HCV-positive individuals, frequencies of sharing of razors (22%) and shaving at the barber shop (22%) were high, followed by tattooing (16.3%), and of them, sharing of razors (OR 0.23, CI: 0.10–0.54, *p*<0.05) and shaving at the barber shop (OR 0.41, CI: 0.22–0.77, *p*<0.05) were statistically significant. In the Juanga tribe, sharing of razors was recorded more commonly in 18.6% HCV-positive individuals, followed by shaving by the village barber (12.4%), among which sharing of razor (OR 0, *p*<0.05) was found to be significantly associated with transmission of HCV. Among females, tattooing (OR 0.527, CI: 0.34–0.80, *p*<0.05) and history of multiple injections (OR 2.36 CI: 1.14–4.90 *p*<0.05) were statistically significant in comparison with other risk factors.

**Table 2.** Distribution of Associated Risk Factors Among Primitive Tribal Groups with Respect to Sero Status of Hepatitis C Virus

	Tribe wise prevalence/distribution											
	Lodha (*n*=242)			Khadia (*n*=450)			Mankidia (*n*=401)			Juanga (*n*=460)		
Associated risk factors	Total No. (%)	HCV +ve (%)	OR (95% CI)	Total No. (%)	HCV +ve (%)	OR (95% CI)	Total No. (%)	HCV +ve (%)	OR (95% CI)	Total No. (%)	HCV +ve (%)	OR (95% CI)
Tattooing	18 (7.4)	0 (0)	1.30 (0)	57 (12.7)	4 (7.02)	1.06 (0.32–3.46)	135 (33.6)	22 (16.30)	0.68 (0.27–1.68)	61 (13.3)	4 (6.56)	0.73 (0.20–2.67)
Sharing of razor	18 (7.4)	2 (11)	0.10 (0.01–1.17)	132 (29.3)	10 (7.58)	0.53 (0.01–33.69)	86 (21.4)	19 (22)	0.23 (0.10–0.54)^a^	61 (13.3)	11 (18)	0.01^a^
Body piercing	62 (25.6)	4 (6.45)	0.14 (0.021–1.01)	193 (42.9)	15 (7.77)	0.36 (0.13–0.92)	181 (45.13)	29 (16)	0.48 (0.19–1.21)	212 (46.1)	11 (5.19)	1.98 (0.81–4.86)
History of multiple injections	56 (23.1)	1 (1.8)	0.40 (0.04–4.20)	274 (60.9)	20 (7.30)	2.63 (0.59–11.65)	241 (60.1)	37 (15.35)	0.96 (0.25–3.68)	247 (53.7)	17 (6.88)	0.09 (0.01–0.61)
Shaving by village barber	77 (31.8)	3 (3.9)	0.75 (0.07–8.21)	133 (29.6)	10 (7.52)	0.71 (0.01–44.58)	86 (21.4)	19 (22)	0.41 (0.22–0.77)^a^	99 (21.5)	12 (12.12)	3.09 (0.36–26.5)
Male	144	4	1.48 (0.36–6.1)	226	12	1.18 (0.53–2.63)	195	27	0.93 (0.52–1.66)	229	26	0.46 (0.23–0.93)^a^
Female	98	4		224	14		206	27		221	13	

^a^ *p*<0.05.

CI, confidence interval; HCV, hepatitis C virus; OR, odds ratio.

Community behavior and sociocultural observation of these two tribes are summarized below, which can be correlated with the identified risk factors. The Mankidia tribe was observed to be nomadic in nature. They gather forest products in conducive seasons and roam around different places in the plain land areas to sell the product, for which they move along with families and live in temporary camps where they come in contact with the nontribal population and get exposed to risk of contact through shaving by the village barber. The traditional, nondisposable steel blades were reported to be still in use. In contrast, the Juanga tribe has been seen to start agricultural labor as an occupation, for which they moved to nearby villages and worked along with the nontribal population as paid laborers. Similar contact with the village barber has been observed.

## Discussion

The study reports the first evidence on HCV prevalence in PTGs from eastern India. The prevalence of HCV infection was 3.7% to 13.4% in five PTGs. In two of these tribes, Mankidia and Juanga, anti-HCV prevalence was seen to be 13.4% and 8.4%, respectively, which is much higher than the infection rate in the general population reported in the country ranging between 0.09% and 0.8%.^1,3,4,15^ Screening of voluntary blood donors has also shown low HCV prevalence of <2%.^2,16^ In this context, PTGs of the region were seen to have a high risk of transmission. In comparison with the available reports of HCV infection in tribal populations, this study shows the highest HCV infection rates among PTGs, except that of 13.7% in tribes of the Northeast.^9^ Tribals from other parts of the country have also shown higher HCV prevalence, that is, 2% in the Lamda tribe of south India (8) and 7.89% in the tribal population of Arunachal Pradesh.^6^

Our study revealed genotype 1b, which was also reported from studies done in eastern and northeastern parts of India, although it is not a common genotype in this region.^8,17–19^

The above report of very high prevalence of infection raises concerns because of the already low population size of PTGs. These populations represent a socially different class with reference to their ancient practices, habits, and rituals and they might have specific risk factors allowing transmission of HCV within the community; however, this has not been adequately addressed. Our study has shown shaving by the village barber and sharing of razors as significant risk factors for transmission of HCV among males in the studied PTGs and in the two tribes (Mankidia and Juanga). Sharing of the blade during shaving, which was observed as a practice, reflects the chance of HCV spread within the community once it is acquired. In case of females, the spread could have been due to transmission during the tattooing or body piercing process. It was observed during the investigation that young women and adolescent girls get tattoos on body parts as a culture of the society. For tattoos, nondisposable metallic needles were used on multiple individuals in a group or in a session without any practice of sterilization or antiseptic use. Similarly, body piercing for the use of ornaments is done by using the same needle. These were presumed to be responsible for contamination with blood products transmitting the virus.

Although the above risk factors are evident from quantitative data recorded from the study, individual general observation of daily activities and social behavior of these two tribes may give some indications toward the high transmission. Total population of Mankidia being restricted to hundreds, marriages happen to be within the tribe in a closed group. However, multiple sexual partners, if any, could not be concluded from the individual or group discussions. The nomadic nature of the tribe has given wide exposure to nontribal modern populations, which allows spread of the infection to naïve PTGs. Being a hunter tribe, they are also frequently affected by injuries, bleeding from sharp weapons such as arrows, and person-to-person contact of blood products with risk of HCV transmission. This was also true for small children who play with sharp objects used for agricultural purposes in case of the Juanga tribe and for hunting purposes in case of the Mankidia tribe. This could have been the possible explanation for high prevalence of HCV infection in young children. In addition, a greater access of the Juanga population to village-level, untrained health practitioners had increased exposure to unsafe injections. As a general belief, these tribes prefer injections over tablets or syrups for better and rapid relief. Therefore, young children having proneness to repeated acute respiratory and gastrointestinal infections had more frequent exposure to unsterile needles.

Females have a fascination toward permanent tattoos, made by needle pricks that result in bleeding from the skin. Especially, adolescent girls get tattooed in groups while enjoying local songs recited by the lady who makes the tattoo. History of injections by multiuse syringes carries a risk for the whole population.

As mentioned above, there is preference for injectable medication for fever, body ache, and exertions, etc., in the form of intramuscular analgesics and intravenous fluids. However, there was no record of injectable drug use in the community. Local alcoholic drinks made from fermented cooked rice and Mohua flower (*Madhuca longifolia*) being the habit of both males and females and the whole village going on festive intoxication during seasonal festivals, premarital sex cannot be ruled out as a mode of HCV transmission, besides the other risks.

Thus, tattoos and injections with unsterile needles, sharing of razors or shaving blades, and unsafe handling of injuries from sharp weapons can be considered as risk factors that are preventable through education and health system interventions.

The herbal remedies they use for treatment of cut wounds and their regular dietary components having a possible medicinal role need to be studied, which can give a clue toward the relatively low disease prevalence even with high seroprevalence for HCV infection.

A recent entry of HCV infection into tribal communities with high transmission risk may be an alternate explanation for high prevalence of infection, but not disease. Any unexplained host protection mechanism following exposure to disease development may also be taken into account. A follow-up of the population for morbidity will be useful in exploring the possibility of HCV infection and diseases associated with chronicity of the infection.

Globally, there are many tribes or communities showing dwindling population growth where causes need to be explored. This report suggests that focus on parenteral infections such as HIV, HBV, and HCV is required, especially looking into sociobehavioral practices in these communities and strategies to reduce transmission through behavioral change.

## Abbreviations Used

CI: confidence interval
HCV: hepatitis C virus
NJ: neighbor-joining
OR: odds ratio
PTGs: primitive tribal groups
